# Intestinal Structure and Function of Broiler Chickens on Diets Supplemented with a Synbiotic Containing *Enterococcus faecium* and Oligosaccharides

**DOI:** 10.3390/ijms9112205

**Published:** 2008-11-12

**Authors:** Wageha Awad, Khaled Ghareeb, Josef Böhm

**Affiliations:** 1 Institute of Nutrition, Department of Veterinary Public Health and Food Science, University of Veterinary Medicine, Veterinärplatz 1, A-1210 Vienna, Austria. E-Mails: ghareebkm@yahoo.com (K. G.); Josef.Boehm@vu-wien.ac.at (J. B.); 2 Department of animal Behaviour and Management, Faculty of Veterinary Medicine, South Valley University Qena, Egypt

**Keywords:** Chicken, synbiotic feed additive, histology, electrophysiological parameter, glucose absorption

## Abstract

A feeding trial was conducted on broiler chickens to study the effects of the synbiotic BIOMIN IMBO [a combination of *Enterococcus faecium*, a prebiotic (derived from chicory) and immune modulating substances (derived from sea algae)], with a dose of 1 kg/ton of the starter diets and 0.5 kg/ton of the grower diets on the intestinal morphometry and nutrient absorption. The general performance was improved (P < 0.05) by the dietary inclusion of synbiotic compared with the controls. Furthermore, the addition of synbiotic increased (P < 0.001) the villus height/crypt depth ratio and villus height in ileum. However, the ileal crypt depth was decreased by dietary supplementation of synbiotic compared with control. The addition of glucose in Ussing chamber produced a significant increase (P ≤ 0.001) in short-circuit current (Isc) in jejunum and colon relative to the basal values in both synbiotic and control groups. However, in jejunum the percentage of Isc increase after glucose addition was higher for synbiotic group (333 %) than control group (45 %). In conclusion, dietary inclusion of synbiotic BIOMIN IMBO increased the growth performance and improved intestinal morphology and nutrient absorption.

## 1. Introduction

Direct-fed microbials (probiotics) have been utilized to improve animal performance by maintaining the normal microflora of host animals. The main action of probiotics is a reinforcement of the intestinal mucosal barrier against deleterious agents [1]. But the prebiotic has been defined as “a non-digestible food that beneficially affects the host by selectively stimulating the growth and/or activity of one or a limited number of bacteria in the intestine” [2–3]. The efficacy of probiotics may be potentiated by the several methods: the selection of more efficient strains; gene manipulation; the combination of several strains; and the combination of probiotics and synergistically acting components. This approach seems to be the best way of potentiating the efficacy of probiotics and is widely used in practice. Synbiotics is defined as a mixture of probiotics and prebiotics that beneficially affects the host by activating the metabolism of one or a limited number of health promoting bacteria and/or by selectively stimulating their growth improving the host’s welfare [2].

Recent research and development of synbiotic products has been increasingly focused on functional benefits including resistance to gastrointestinal bacterial infection, antibacterial activity, and improved immune status in broiler chicks. Zhang *et al*. [4] found that some probiotics or synbiotics were effective in increasing the body weight of chickens. In addition, Mohnl *et al*. [5] found that the synbiotic product (Biomin® PoultryStar) had a comparable potential to improve broiler performance as Avilamycin (an antibiotic growth promoter). It seems that synergistic effects of prebiotics and probiotics can be useful in stimulating beneficial bacteria and improving the health of the gut. However, there is scarce information available to date on synbiotics and its possible mechanisms in broiler chickens.

*Enterococci* are among the wide variety of microbial species that have been used extensively as probiotics [6]. Following feeding of probiotics, improvements in growth performance and feed efficiency have been reported in broiler chickens [7–12]. Recently, it was shown that adding of probiotic containing *Enterococcus faecium* microorganism to broiler diets increased the jejunal villus height [13]) and ileal villus height [11]. Moreover, increased intestinal villi height was reported after addition of *Bacillus subtilis* in association with prebiotics [15].

It is assumed that an increased villus height is paralleled by an increased digestive and absorptive function of the intestine due to increased absorptive surface area, expression of brush border enzymes and nutrient transport systems [16]. It is well known that many substances can affect the intestinal villi development. Enterocyte enzymatic activity and structure are two of the most important features of the intestinal mucosal physiology. However, no information is available regarding the effect of adding synbiotic product to broiler diets on the intestinal morphology and *in vitro* absorptive capacity of glucose across the isolated intestinal mucosa. Based on this concept, the goal of the present study was to investigate the effects of synbiotic product (Biomin^®^ IMBO) on broiler performance, the intestinal morphology and the electrical properties of intestinal mucosa of broiler chickens.

## 2. Materials and Methods

### 2.1. Birds, Housing and Diets

Four hundred, 1-day-old broiler chicks (males and females) were obtained from a commercial hatchery. The birds were weighed at the beginning of the experiment, randomly divided without regard to sex into two groups (200 birds/group), and housed in pens of identical size (1.75 × 6 m) in a deep litter system. Wood shavings were used as the litter material. Each group had eight replicates (25 birds/pen). The climatic conditions and lighting program were computer-operated and followed the commercial recommendations. Environmental temperature in the first week of life was 35 °C, and decreased to 25 °C till the end of experiment. During the first week 22 hours of light were provided with a reduction to 20 hours afterwards.

The control group was fed starter and grower diets based on corn, soya HP, soya oil, and a premix with vitamins, minerals, amino acids (lysine, methionine, threonine), salt and monocalcium phosphate. The synbiotic group was fed the basal diet plus the synbiotic product (1 kg of Biomin^®^ IMBO/ton of the starter diets and 0.5 kg/ton of the grower diets). Biomin® IMBO is a combination of probiotic strain *Enterococcus faecium* (DSM 3530), added to the starter diet with 5 × 10^8^ cfu/kg and 2.5 × 10^8^ cfu/kg to the grower diet, prebiotic derived from chicory rich in inulin, and immune-modulating substances derived from sea algae. The chicks were fed with the starter diets from days 1 to 13 and grower feed from day 14 – 35 (Table 1). The feed additives were delivered by Biomin® GmbH, Herzogenburg, Austria. The birds had free access to water and feed.

### 2.2. Traits

#### 2.2.1. Performance and morphology of the intestinal tract

Chicks were weighed individually at the beginning of the experiment (initial body weight) as well as at the end of feeding period (at day 35). The feed consumption was measured weekly during the 5-wk experiment. Cumulative weight gain; feed consumption and food conversion ratio (food intake/weight gain, FCR) were calculated. The mortality % and liveability % at the end of the feeding period were determined. The European Production Efficiency Factor (EPEF) was calculated according to the following equation: EPEF= liveability % x live weight (kg) / age (d) × FCR × 100.

At the end of the feeding period, 10 birds from each group were randomly selected, and killed by cervical dislocation. The whole intestinal tract was removed and before removal of the content, segments from the duodenum (the midpoint), and ileum (10 cm proximal to the ileocecal junction) were taken. Segments were fixed in 10% neutral buffered formalin solution and embedded in paraffin wax. All histological studies were performed on 5 μm sections (4 cross-sections for each sample), stained by haematoxylin and eosin, and examined by Olympus AX70 microscope (Olympus Cooperation, Tokyo, Japan) fitted with a digital video camera (Sony DXC-930P).

The total of the intact well-oriented, crypt-villus units were selected in triplicate for each intestinal cross-section for each sample. The criterion for villus selection was based on the presence of intact lamina propria. The villus length was measured from the villus tip to the villus-crypt junction, while crypt depth was defined as the depth of the invagination between 2 villi. The measurement was done with the stereological image software, Cast Image System (Version 2.3.1.3) (Visiopharm, Horsholm, Denmark). The mean villus heights and crypt depth from 10 birds were expressed as a mean villus height for 1 treatment group.

#### 2.2.2. Electrophysiological Parameters and Nutrient Transport Activity

At the end of the feeding period, five birds (six replicates/bird) from each group were killed, and the basal and glucose stimulated transmural potential difference (PD), short-circuit current (Isc), and electrical tissue conductance (Gt) were measured in the isolated gut mucosa to characterize the electrical properties of the gut.

Immediately after exsanguinations, segments were taken from the mid-jejunum and colon. Segments from colon were taken to be representative for the effect of synbiotic in the large intestine, because of its higher absorptive capacity in chickens as shown by Amat *et al.* [16], in addition, the synbiotic effect may be directed towards both the small and the large intestines.

After preparation of stripped intestinal sheets (removal of serosal layer), the tissue was mounted in modified Ussing chambers with an active area of 1 cm^2^. The serosal and mucosal surfaces of the tissues were bathed in 5 mL of Ringer solution with the following composition (mmol/L): CaCl_2_, 1.2; MgCl_2_, 1.2; Na_2_HPO_4_, 2.4; NaH_2_PO_4_, 0.4; NaHCO3, 25; KCL, 5; NaCl, 115; mannitol, 20 for the serosal side. Ringer solution was added to mucosal side and 5 mmol d-glucose was added instead of mannitol. The pH of the solution was adjusted to 7.4 using a pH meter. The incubation medium was continuously gassed with a mixture of 95% O_2_ and 5% CO_2_, and the temperature of the mixture was kept at 38 °C. Continuous oxygenation provided recirculation of the incubation solutions by means of a gas lift.

The tissues were first incubated under open circuit conditions for 20 min and then voltage clamped by fixing the voltage at 0 mV. The electrophysiological parameters (Isc and Gt) were recorded. Thereafter, the d-glucose was added at the mucosal side (final concentration: 5 mmol/L). The electrical response to glucose was measured as the peak response obtained approximately 1 min after addition of the solution. The basal Isc is expressed as actual values, whereas the effect of d-glucose to the mucosal side on the electrical variables is shown as the amount of change.

### 2.3. Statistics

Statistical analyses were conducted with SPSS Version 15 (SPSS GmbH, Munich, Germany) to determine if variables differed between groups. The Kolmogorov-Smirnov test was used to test the normal distribution of the data before statistical analysis was performed. Results are expressed as means ± pooled SEM. For body weight gain, feed intake, feed conversion, intestinal morphological parameters, independent samples *t*-test was used between the 2 groups. For electrical parameters (nutrient transport study), a paired *t*-test was used for the comparison of the different measurements within the same group. The independent samples *t*-test was used to compare the electrical parameters between the 2 groups. Probability values of less than 0.05 (*P* < 0.05) were considered significant.

## 3. Results

The supplementation of synbiotic (Biomin^®^ IMBO) to the diets significantly improved the general performance of broilers (Table 2). The mean body weight over the course of the experiment was higher (P < 0.05) for synbiotic group (1,847 g) compared with controls (1,754 g). The average daily weight gain was higher (P < 0.05) for broilers fed the diet supplemented with synbiotic (52 g) compared with the control birds (49 g). The food conversion ratio was lower for birds supplemented with synbiotic (1.75) than control birds (1.89). Additionally, the European Production Efficiency Factor (EPEF) was higher for synbiotic supplemented group (291) than control group (255).

The histological study showed that the addition of synbiotic increased (P < 0.001, Table 3) the villus height/crypt depth ratio and villus height in ileum compared with controls. However, the ileal crypt depth was decreased by synbiotic supplementation (117 ± 2 μm) compared with control (128 ± 2 μm).

The addition of 5 mmol d-glucose/L on the mucosal side of Ussing chamber produced a significant increase in Isc (P ≤ 0.001, Table 4) in jejunum and colon in both groups relative to the basal values. Moreover, in jejunum the actual basal Isc and Isc after d-glucose addition values were lower for synbiotic group compared with the control group (P < 0.05). However, the amount of Δ Isc after d-glucose addition was equally similar for the 2 groups. Interestingly, the percentage of Isc increase after d-glucose addition (ΔIsc %) was higher in synbiotic group (333%) than control group (45%).

Moreover, the conductance of colonic tissues remained unaffected by the dietary supplementation of synbiotic as there was no significant difference (P > 0.05) between the groups (Table 5). The basal conductance of jejunal mucosa was lower for the synbiotic group compared with control group (P< 0.05).

## 4. Discussion

In the recent decades, deficiencies in feed formulation and management practices have been masked by the routine use of antibiotic growth promoters (AGP). However, the ban of AGP in Europe has driven the implementation of alternative strategies in order to maintain health and performance status and optimising digestion in poultry production. Several feed additives have been used to manipulate microbial communities in the digestive tract. However, their efficacy has not always been proven and their modes of action require further research. The present study focused on the role and the efficacy of the synbiotic product as potential modulators of gut health and growth performance in poultry production.

Probiotics have been reported to improve microbial balance in the gastrointestinal tract through bacterial antagonisms, competitive exclusion and immune stimulation. Prebiotics which include non-digestible oligosaccharides may control or manipulate microbial composition and/or activity, thereby assisting to maintain a beneficial microflora that suppresses through different regulatory mechanisms the growth of pathogens. The combination of probiotics and prebiotics, also referred to as synbiotics, may improve the survival rate of probiotics during their passage through the digestive tract, thus contributing to the stabilisation and/or enhancement of the probiotic effects.

The results of the present feeding trial provide evidence that the dietary inclusion of synbiotic (Biomin IMBO) improved (P < 0.05) the body weight and body weight gain of broiler chickens. The average daily weight gain was significantly higher for broilers fed the synbiotic diet compared with the broilers fed the control diet. This indicates that the synbiotic can be used as a growth promoter in broiler diets. These products show promising effects as alternatives for antibiotics as pressure to eliminate growth promotant antibiotic use increases.

The present study shows changes in the mucosal architecture in terms of increased ileal villus height to crypts depth in birds fed with synbiotic supplemented diet. The intestinal mucosal architecture can reveal useful information on the intestinal function. Increasing the villus height suggests an increased surface area capable of greater absorption of available nutrients [18]. Samanya and Yamauchi [19] found that longer villi in the ileum of adult male layer with slight improvement in feed efficiency after dietary addition of *Bacillus subtilis var. natto* and in broilers after addition Enteroccocus faecium [11] or Eubacterium sp. [20].

The intestinal microbiota plays a vital role in the normal nutritional, physiological, immunological, and protective functions of the host animals [21]. The composition and metabolic activity of the intestinal microbiota can be influenced by the diet [22]. There is a growing interest in the use of a variety of probiotics and synbiotics to promote animal health by altering the intestinal microbial community. Although a marked proportion of the beneficial effects of probiotics or synbiotic so far discussed seem to be attributable to certain epithelial function, there are relatively few experimental data to support this hypothesis.

In chicken, it was shown that addition of d-glucose increased the Isc (short circuit current) in all intestinal segments including duodenum, jejunum, ileum, and colon due to increased Na^+^ d-glucose co-transport [23] and the effect is maximal in colon. The effect of addition of d-glucose on the electrophysiological properties of intestinal mucosa of broilers fed a synbiotic was not studied yet. Therefore, the present experiment investigated the effect adding synbiotic (Biomin IMBO) on the electrophysiological parameters of intestinal mucosa of broiler chicken.

The small and large intestines of birds are known to have high absorptive rates for water and electrolytes. The movement of ions responsible for the electrical current across the epithelium are mainly result of the absorption of Na^+^ and the secretion of Cl^−^ [24, 25].

In the present study, the addition d-glucose produced a significant increase in Isc in both jejunum and colon in both groups relative to the basal values. However, the basal Isc and Isc value after glucose addition in jejunum was lower for birds fed with synbiotic compared with the controls. The lower and negative basal Isc value in synbiotic group may indicate that the synbiotic has a proabsorptive effects for water and electrolytes through stimulation of trans- and paracellular diffusional absorption. As it was shown that increased absorption of glucose after feeding of probiotic is linked to increase passive absorption of glucose and facilitate its transport in the gastrointestinal tract [26]. Another possible explanation for the effect of synbiotic on the basal current is their antisecretory Cl^−^ effect, which may help to explain the reduce incidence of diarrhoea upon treatment with direct fed microbials [27]. Moreover, it was shown that addition of direct fed microbial, *Streptococcus faecium* C-68 decreased the E. coli induced diarrhoea in gnotobiotic pigs [28]. Similarly, Krammer and Karbach [29] have reported an antidiarrheal effect of the probiotic *Saccharomyces boulardii* in the rat small intestine by stimulating chloride absorption. The addition of d-glucose produced a higher ΔIsc % in the synbiotic group (333%) than control group (45%). The results reported here indicate that dietary synbiotic can influence electrophysiological parameters of the gut, indicating that the addition of synbiotic to broiler feed increased the intestinal glucose absorption. This result is in agreement with Lodemann *et al*. [27] who found that the dietary inclusion of *Enterococcus faecium* increased the glucose absorption in piglet. This suggests that the addition of chicory and other compound to *E. faecium* plays a little role in the beneficial effect of synbiotic. As it was shown that inulin supplementation did not modify the electrogenic transport of glucose [30].

Moreover, the basal conductance of jejunal mucosa was decreased by the dietary inclusion of synbiotic (P < 0.05). The tissues conductance after d-glucose addition remained unchanged by dietary synbiotic which is in agreement with Winckler *et al.* [31] who reported that the tissue conductance after glucose addition remained unaffected in the jejunum of pigs after feeding of *S. boulardii*. Contrary to that, Breves *et al.* [32] who reported an increase in the tissue conductance after glucose addition in the jejunal epithelia from probiotic *S. boulardii* or *Bacillus cereus* var fed pigs. The regulation of intestinal transport functions mainly depends on the junctional complex connecting enterocytes together [33] that also determines the extent to which solutes and water are absorbed or secreted. As a barrier between the luminal and basolateral compartments, tight junctions selectively control the passive diffusion of ions and other small solutes through the paracellular pathway and thereby influence any gradient created by the activity of pathways associated with the transcellular route. The presence of paracellular pathways with high permeability, such as in the small intestine, permits rapid transepithelial diffusion and precludes the presence of a large transepithelial electrical potential and facilitates the transport of antigenic and toxic substances across the intestinal mucosa. Therefore, the result reported here indicating that addition of synbiotic to broiler diet enhanced the maintenance and function of the epithelial barrier.

## 5. Conclusions

In conclusion, from this study we can conclude that synbiotic can exert beneficial effects in the gastrointestinal tract as a result of alteration in whole body, feed consumption, and absorption of nutrient and beneficial changes in intestinal architecture. But knowledge of the plausible interactions between food contaminants and natural components has not yet been studied. Therefore, future studies should address the question of whether natural feed additives such as probiotics and synbiotics would attenuate the toxic effect of mycotoxins at the gut level.

## Figures and Tables

**Table 1.** Composition of the basal experimental diet (%).

Ingredient	Starter	Grower
Corn	57.93	59.75
Soya HP	31.25	29.60
Soya oil	2.50	2.00
Megafat	1.25	2.50
Monocalciumphosphate	0.25	
Lysine	0.38	0.15
Methionin	0.08	
Threonine	0.13	
Premix^a^	6.25	6.00
Calculated composition b		
Dry matter	88.7	89.1
Crude protein	22.1	21.5
ME (MJ/kg)	14.28	14.66
Crude fat	7.6	8.4
Ca	1.56	1.41
P	0.97	0.83
Na	0.30	0.28
Mg	0.32	0.28

a BR 5 Universal Vetmed, Biomin GmbH, Herzogenburg, Austria. Each kg contains calcium 196 g, phosphorous 64 g, sodium 30 g, magnesium 6 g, copper 400 mg, zinc 1,200 mg, iron 2,000 mg, manganese 1,200 mg, cobalt 20 mg, iodine 40 mg, selenium 8 mg, vitamin A 200,000 IU, vitamin D_3_ 80,000 IU., vitamin E 1,600 mg, vitamin K_3_ 34 mg, vitamin C 1,300 mg, vitamin B_1_ 35 mg, vitamin B_2_ 135 mg, vitamin B_6_ 100 mg, vitamin B_12_ 670 mcg, nicotinic acid 1,340 mg, calcium pantothenic acid 235 mg, choline chloride 8,400 mg, folic acid 34 mg, biotin 3350 μg, methionine 30g.

b Based on a dry matter content of 88 %.

**Table 2.** Effects of dietary inclusion of synbiotic on performance of broilers.

	Dietary treatment			
Parameters	Control	Synbiotic	SEM	P Value
Initial body weight	40.32	40.29	0.2	0.955
Final Body Weight at day 35	1754 ^b^	1847 ^a^	8	0.001
Daily weight gain	49 ^b^	52 ^a^	0.2	0.001

Within the same row means with different superscripts are significantly different (Independent t-test). (n = 200/treatment).

**Table 3.** Effects of dietary inclusion of synbiotic on the intestinal morphological parameters of broilers.

	Dietary treatment			
Parameters	Control	Synbiotic	SEM	P Value
1- Duodenum				
Villus height (μm)	1,640	1,647	14	0.823
Crypt depth (μm)	149	149	2	0.873
Villus height/crypt depth	11.45	12.00	0.2	0.135
2- Ileum				
Villus height (μm)	614 ^b^	774 ^a^	9	0.001
Crypt depth (μm)	128 ^a^	117 ^b^	1	0.001
Villus height/crypt depth	4.86^b^	7.13^a^	0.1	0.001

Within the same row means with different superscripts are significantly different (Independent sample t-test). (n = 10/treatment).

**Table 4.** Effects of dietary inclusion of synbiotic on short-circuit current (Isc) (μA/cm^2^) in isolated jejunal and colonic mucosa of broilers after glucose addition.

	Dietary treatment			
Parameters	Control	Synbiotic	SEM	P Value
1- Jejunum				
Basal Isc (μA/cm^2^)	22^a^	−3^b^	6	0.038
Isc after glucose addition(μA/cm^2^)	32^a^	7^b^	6	0.030
Δ Isc^1^ (μA/cm^2^)	10	10	1	0.955
2- Colon				
Basal Isc (μA/cm^2^)	7	11	19	0.942
Isc after glucose addition(μA/cm^2^)	46	47^a^	17	0.994
Δ Isc^1^ (μA/cm^2^)	39	38	7	0.961

Within the same row means with different superscripts are significantly different (Independent samples t-test). (n = 5/treatment).

**Table 5.** Tissue conductance (Gt, mS/cm^2^) across the isolated jejunal and colonic mucosa of broilers after glucose addition.

	Dietary treatment			
Parameters	Control	Synbiotic	SEM	P Value
1- Jejunum				
Basal Gt (mS/cm^2^)	2.87 ^a^	2.30 ^b^	0.3	0.036
Gt after glucose addition (mS/cm^2^)	2.86	2.33	0.4	0.131
2- Colon				
Basal Gt (mS/cm^2^)	8.27	6.69	0.6	0.316
Gt after glucose addition (mS/cm^2^)	7.97	6.56	0.7	0.388

Within the same row means with different superscripts are significantly different (Independent samples t-test). (n = 5/treatment).
